# Whole-Genome Sequence of Infectious Pancreatic Necrosis Virus Isolated from Farmed Brook Trout (Salvelinus fontinalis) in Pennsylvania

**DOI:** 10.1128/genomeA.00360-18

**Published:** 2018-04-26

**Authors:** Kay Palchak, Shubhada K. Chothe, Aswathy Sebastian, Ruth H. Nissly, Rhiannon Barry, Istvan Albert, Bhushan M. Jayarao, Suresh V. Kuchipudi

**Affiliations:** a Penn State Animal Diagnostic Laboratory, Department of Veterinary and Biomedical Sciences, Pennsylvania State University, University Park, Pennsylvania, USA; b Department of Biochemistry and Molecular Biology, Pennsylvania State University, University Park, Pennsylvania, USA

## Abstract

Infectious pancreatic necrosis (IPN) is an acute contagious systemic disease affecting several fish species and a critical disease in the salmonid fish farming industry. Here, we report the complete genome sequence of IPN virus (IPNV) RNA segments A and B, isolated from a farmed brook trout in Pennsylvania.

## GENOME ANNOUNCEMENT

Infectious pancreatic necrosis (IPN) is a highly contagious viral disease of salmonid fish caused by infectious pancreatic necrosis virus (IPNV). IPNV belongs to the genus Aquabirnavirus of the family *Birnaviridae*. This prototype virus of the *Birnaviridae* family is nonenveloped and measures approximately 65 nm in diameter with icosahedral symmetry. The IPNV genome consists of two linear double-stranded RNA segments which are designated segments A and B. Segment A is bicistronic and encodes a 106-kDa polyprotein (NH2-pVP2-VP4-VP3-COOH), whereas segment B is monocistronic and encodes the RNA-dependent RNA polymerase VP1 (1). IPNV is a major viral pathogen in salmon and is the most common pathogen in the aquatic fauna (2).

Along with several salmonid fish species, other types of fish, such as rainbow and brook trout, are also susceptible to IPNV. The IPNV infection manifests as swelling of the abdomen, bilateral exophthalmos, and pale gills. The infection is subclinical in fish older than 4 months, and the mortality rate ranges from 10 to 90% (3). Here, we report the whole-genome sequence of IPNV isolated from a farmed brook trout (*Salvelinus fontinalis*) and submitted to the Animal Diagnostic Laboratory, Pennsylvania State University. Kidney and spleen tissues from the brook trout were homogenized in viral transport medium (VTM) and inoculated in Chinook salmon embryo (CHSE-214) cells grown at 18°C in minimal essential medium supplemented with 2 mM glutamine, 1% nonessential amino acids, and 1× penicillin, streptomycin, and amphotericin B for virus isolation. After three freeze-thaw cycles, the infected viral supernatant and the lysed CHSE-214 cells were centrifuged at 1,500 × *g* for 10 min. The resulting supernatant was used for viral RNA extraction using a QIAamp viral RNA minikit (Qiagen, Germantown, MD, USA).

Using the Illumina TruSeq stranded mRNA kit, a barcoded library was prepared from the IPNV viral RNA. The manufacturer’s protocol was followed using the “purified mRNA input” recommendation, which skips poly(A) RNA enrichment and begins with RNA fragmentation and priming. An equimolar pool of libraries was made and sequenced on an Illumina MiSeq instrument using 150 × 150-bp paired-end sequencing according to the manufacturer’s protocol. Over 611,110 reads were generated for the IPNV genome. Low-quality bases were trimmed by Trimmomatic (4) using a sliding-window approach. Trimmed reads were mapped to a reference (NCBI reference sequence numbers NC_001915 and NC_001916) using the Burrows-Wheeler Aligner MEM algorithm (BWA-MEM) (5) and visualized in the Integrative Genomics Viewer (IGV) (6). The mapped reads were then assembled using the SPAdes (v3.11.0) (7) *de novo* assembler to produce two RNA segments. The IPNV consensus sequences of segments A and B were annotated with Prokka (v1.12) (8).

### Accession number(s).

RNA segments A and B of isolate PA1 were submitted to GenBank and assigned the accession numbers MH010544 and MH010545, respectively.
